# Mutations of *C19orf12*, coding for a transmembrane glycine zipper containing mitochondrial protein, cause mis-localization of the protein, inability to respond to oxidative stress and increased mitochondrial Ca^*2+*^

**DOI:** 10.3389/fgene.2015.00185

**Published:** 2015-05-19

**Authors:** Paola Venco, Massimo Bonora, Carlotta Giorgi, Elena Papaleo, Arcangela Iuso, Holger Prokisch, Paolo Pinton, Valeria Tiranti

**Affiliations:** ^1^Unit of Molecular Neurogenetics – Pierfranco and Luisa Mariani Center for the study of Mitochondrial Disorders in Children, IRCCS Foundation Neurological Institute “C. Besta”Milan, Italy; ^2^Section of Pathology, Oncology and Experimental Biology and Laboratory for Technologies of Advanced Therapies Center, Department of Morphology, Surgery and Experimental Medicine, University of FerraraFerrara, Italy; ^3^Structural Biology and NMR Laboratory, Department of Biology, University of CopenhagenCopenhagen, Denmark; ^4^Institute of Human Genetics, Klinikum rechts der Isar, Technische Universität MünchenMunich, Germany; ^5^Institute of Human Genetics, Helmholtz Zentrum MünchenMunich, Germany

**Keywords:** mitochondria, oxidative stress, neurodegeneration with brain iron accumulation, endoplasmic reticulum-mitochondria associated membranes (ER-MAM), molecular modeling, simulation

## Abstract

Mutations in *C19orf12* have been identified in patients affected by Neurodegeneration with Brain Iron Accumulation (NBIA), a clinical entity characterized by iron accumulation in the basal ganglia. By using western blot analysis with specific antibody and confocal studies, we showed that wild-type C19orf12 protein was not exclusively present in mitochondria, but also in the Endoplasmic Reticulum (ER) and MAM (Mitochondria Associated Membrane), while mutant C19orf12 variants presented a different localization. Moreover, after induction of oxidative stress, a GFP-tagged C19orf12 wild-type protein was able to relocate to the cytosol. On the contrary, mutant isoforms were not able to respond to oxidative stress. High mitochondrial calcium concentration and increased H_2_O_2_ induced apoptosis were found in fibroblasts derived from one patient as compared to controls. C19orf12 protein is a 17 kDa mitochondrial membrane-associated protein whose function is still unknown. Our *in silico* investigation suggests that, the glycine zipper motifs of C19orf12 form helical regions spanning the membrane. The N- and C-terminal regions with respect to the transmembrane portion, on the contrary, are predicted to rearrange in a structural domain, which is homologs to the N-terminal regulatory domain of the magnesium transporter MgtE, suggesting that C19orf12 may act as a regulatory protein for human MgtE transporters. The mutations here described affect respectively one glycine residue of the glycine zipper motifs, which are involved in dimerization of transmembrane helices and predicted to impair the correct localization of the protein into the membranes, and one residue present in the regulatory domain, which is important for protein-protein interaction.

## Introduction

The acronym NBIA identifies a group of clinically and genetically heterogeneous rare pathological conditions, characterized by progressive extra-pyramidal disorders and by evidence of focal iron accumulation in the brain, especially in basal ganglia, and globus pallidus, observed in MRI studies.

Recently, thanks to the identification of new disease genes in these years there has been an increasing knowledge about NBIA, but pathomechanisms underlining these disorders are still not completely clear. Up to now 10 genes have been associated with specific forms of NBIA (Kalman et al., 2012). Only two forms inherited as autosomal dominant and recessive traits respectively are caused by mutations in genes coding for proteins directly involved in iron metabolism: neuroferritinopathy due to ferritin light chain gene (*FTL)* (MIM#606159) mutation (Chinnery et al., 2007) and aceruloplasminemia linked to mutations in the ceruloplasmin gene (CP) (MIM#117700) (McNeill et al., 2008).

The other forms with autosomal recessive or X-linked transmission are due to mutations in genes (Rouault, 2013) coding for proteins with a variety of functions including: Coenzyme A biosynthesis, fatty acid metabolism, autophagy, and still unknown roles.

This is the case for the *C19orf12* gene, coding for a mitochondrial membrane protein, which mutations are responsible for a form of disease called MPAN for Mitochondrial membrane Protein Associated Neurodegeneration (Hartig et al., 2012). Mean age at onset is 9 years and the clinical phenotype is characterized by: progressive spastic para and tetraparesis, generalized dystonia, optic atrophy, motor axonal neuropathy, and psychiatric signs. T2-weighted MRI reveals hypointensities in the globus pallidus and substantia nigra. Mutations of *C19orf12* were also found in a patient with Parkinson disease (Hartig et al., 2012) and post mortem examination of the brain of one MPAN patient revealed Lewy bodies, tangles, spheroids, and tau pathology, indicating a possible overlap between NBIA and more common neurodegenerative diseases. There is no direct link between *C19orf12* mutations and the clinical phenotype of the patients, although preliminary evidence suggests for this gene a role in lipid homeostasis (Hartig et al., 2012). Recently, a Drosophila model (Iuso et al., 2014) has been generated, which shows neurological problems that can resemble the clinical features present in patients.

To gain insight into the functional properties of wild-type and mutant encoded proteins, corresponding to homozygous mutations Q96P and G58S, identified in two affected patients (Panteghini et al., 2012), we performed immunolocalization and confocal assays under normal and stress conditions. Since no structural information are available on *C19orf12*, we also exploited molecular modeling techniques and we predicted that the protein has transmembrane helices with glycine-zipper motifs and a soluble domain that is homologous to the N-regulatory domain of bacterial MgtE transporter. The mutations identified in the patients are predicted to structurally destabilize both the glycines of the transmembrane zipper motif and the soluble domain, where the Q96P especially may impair the helical structure of the fourth α-helix of the homology model, which correspond to helix α6 of the bacterial domain.

## Methods

### Cloning procedures and plasmid vectors mutagenesis

Human *C19orf12* was cloned in the pCMV-AC-GFP (OriGene) vector containing a C-terminal green fluorescent protein. cDNA was amplified by PCR from pCMV-AC-GFP construct with primers carrying c-myc tag (underlined sequence) described below, and cloning in the pcDNA3.1(-), in order to obtain a recombinant protein with a smaller tag than the GFP-one. The cDNA was PCR amplified with these primers:

Fw: 5′-TCTGCCGCCGCGATCGCCATGGAGA-3′

Rv: 5′-CGGTTATCACAAGTCCTCTTCAGAAATGAGCTTTTGCTCGTCATCATACTGGATCTCGG-3′

The mutant versions corresponding to the G58S and Q96P were obtained by site directed mutagenesis (QuikChange II Site-Directed Mutagenesis Kit Stratagene). The corresponding modified primers used to generate mutated allele are as follows:

G58S Fw: 5′-GGGGGTTTGGTGGGCAGCCCACCGGGACTCGCC-3′

G58S Rv: 5′-GGCGAGTCCCGGTGGGCTGCCCACCAAACCCCC-3′

Q96P Fw: 5′-CCCCCTGCCGAGCCACAGAGGCTCTTTAACGAAGCC-3′

Q96P Rv: 5′-GGCTTCGTTAAAGAGCCTCTGTGGCTCGGCAGGGGG-3′

We use also a vector containing the mkate2 red fluorescent protein (Envrogen) additionally to the GFP in order to perform live imaging experiments. Cloning Procedures and Plasmid Vectors pmKate2-N-c19orf12 was obtained as follows. The two original plasmids pCMV6-AC-GFP and pmKate2-N contained appropriate restriction sites to allow cloning in the EcoRI-XhoI for the first one and EcoRI-SalI for the second one. XhoI and SalI produce compatible cohesive ends and produce recleavable ligation products. All cloned fragments were sequenced to check the absence of mutations. Restriction-enzyme digestions, *Escherichia coli* transformation, and plasmid extractions were performed with standard methods.

### Cell culture, transient transfection, stable transduction

HeLa and HEK-293 cells were grown in Dulbecco's modified Eagle's medium (DMEM) (Euroclone), supplemented with 10% fetal bovine serum (FBS). Cells were seeded 36 h before transfection onto round glass coverslips for imaging or 13-mm diameter petri dishes for aequorin experiments, or in 10-cm petri dishes for immunoblot and fractionation experiments. Cells were allowed to grow to 50% confluence, then transfected with a standard calcium phosphate procedure (Sambrook and Russell, 2006) and used in the experiments 36-h post-transfection.

### Quantitative colocalization analysis

HeLa cells were co-transfected with wild-type or mutant C19orf12 fused in frame with mkate2 fluorescent marker and with the ER marker GFP–Sec61-β. Thirty six hour after transfection, cells were stained with the mitochondrial dye Mitotracker Deep Red 200 nM in PBS for 10 min at 37°. After washing cells were imaged with and LSM510 confocal microscope equipped with a Plan-Apochromat 63X/1.4 n.a. Oil objective and acquired with a pixel size of 142 nm.

### Live imaging

HeLa cells were co-transfected with GFP-tagged C19orf12 wild-type or mutant chimeras and the mitochondrial marker mtDsRed using calcium phosphate method. Thirty six hour after transfection, time-lapse recording were performed with a Nikon Swept Field Confocal equipped with CFI Plan Apo VC60XH objective (numerical aperture, 1.4) (Nikon Instruments, Melville, NY, USA) and an Andor DU885 electron multiplying charge- coupled device (EM-CCD) camera (Andor Technology Ltd, Belfast, Northern Ireland), the overall image sampling was below the resolution limit (X and Y pixel size: 133 nm). Coverslips were placed in an incubated chamber with controlled temperature, CO_2_ and humidity; images were then acquired with a differential frequency during the experiment: cells were placed in 1 mM Ca^2+^ KRB and basal fluorescence images were acquired for 5 min; then cells were stimulated with H_2_O_2_ (500 μM final), and fluorescence images were acquired for 1 h and 30 min.

### Image analysis

Acquired images were then analyzed by using open source software Fiji. Images were corrected for spectral bleedthrough using the Spectral Unmixing plugin (available at http://rsbweb.nih.gov/ij/plugins/spectral-unmixing.html). Then, single cells were analyzed, and, for each of those, the Manders' overlap coefficient was obtained using the JACOP plugin (available at http://rsb.info.nih.gov/ij/plugins/track/jacop.html).

### Mitochondria preparation and fractionation

Isolated mitochondria from cultured cells were obtained according to the protocol described (Fernandez-Vizarra et al., 2010).

Isolated mitochondria were resuspended in 100 ml of potassium phosphate buffer [(PP) buffer, 20 mM, pH 7.8, KCl 150 mM] and sonicated 10 s for three times at 10 Amp. The suspension was centrifuged at 164000 g for 30 min at 48C. Supernatant (mitochondrial matrix and inter-membrane space) was collected, and pellet (mitochondrial membranes) was resuspended in 100 ml of PP buffer.

### MAM and ER fraction preparation

Hek cells (Wieckowski et al., 2009) were harvested, washed in phosphate-buffered saline medium, pelleted by centrifugation at 500 × g for 5 min, resuspended in homogenization buffer (0.25 M sucrose and 10 mM Hepes pH 7.4) and gently disrupted by dounce homogenization. The homogenate was centrifuged twice at 600 × g for 5 min to remove cellular debris and nuclei, and the supernatant was centrifuged at 10.300 × g for 10 min to pellet crude mitochondria. The resultant supernatant was centrifuged at 100.000 × g for 1 h in a Beckman 70 Ti rotor at 40C to pellet microsomes, which were resuspended in homogenization buffer. The mitochondrial pellet, resuspended in isolation medium (250 mM mannitol, 5 mM Hepes (pH7.4), and 0.5 mM EGTA) was layered on top of 8 ml of Percoll medium [225 mM mannitol, 25 mM Hepes (pH 7.4), 1 mM EGTA, and 30% Percoll (v/v)] in a 10-ml polycarbonate ultracentrifuge tube and centrifuged for 30 min at 95.000 × g. A dense band containing purified mitochondria, recovered approximately 3/4 down the tube, was removed, diluted with isolation medium, washed twice by centrifugation at 6.300 × g for 10 min to remove the Percoll, and finally resuspended in isolation medium. MAM, removed from the Percoll gradient as a diffuse white band located above the mitochondria, were diluted in isolation medium and centrifuged at 6.300 × g for 10 min. The supernatant containing MAM was centrifuged at 100.000 × g for 1 h in a Beckman 70 Ti rotor, and the resulting pellet was resuspended in the homogenization buffer.

The quality of the preparation was checked by western blot analysis using different markers for the fractions obtained: IP3R was used as marker of ER, tubulin as marker of cytoplasm and the Voltage Dependent Anion Channel (VDAC) as marker for mitochondria.

### Immunoblot and immunocytochemistry analysis

Thirty micrograms of proteins were used for each sample in denaturing sodium dodecyl sulfate–polyacrylamide gel electrophoresis (SDS–PAGE). Western blot analysis was performed as described (Tiranti et al., 1999), using the ECL-chemiluminescence kit (Amersham) according to the manufacturer's protocol.

### Antibodies

For immunodetection of the C19orf12 protein, western-blot analysis with a antisera specific for C19orf12 (1:1000) was performed, as previously described (Hartig et al., 2012). An anti-Myc monoclonal antibody (OriGene) was used at a final concentration of 1μg/ml. An anti-NADH dehydrogenase ubiquinone 1 alpha subcomplex subunit 9 (NDUFA9) antibody was used (Invitrogen) at final concentration of 0.5 μg/ml. A mouse monoclonal anti-b-TUBULIN antibody was used at a final concentration of 1 μg/ml (Sigma-Aldrich). An anti-ethylmalonic encephalopathy 1 rabbit polyclonal antibody was used at 1:2000 dilution (Tiranti et al., 2004). An anti-VDAC (1:3000) from Abcam (Cambridge, UK). An anti-IP3R3 (1:300) from BD Biosciences (San Jose, CA, USA). Secondary anti-rabbit and anti-mouse antibodies were used at 1:7000 and 1:5000 dilutions, respectively.

### Automated nuclei count analysis

Fibroblasts were seeded at 50,000 cells on a 25-mm coverslip, allowed to grow for 48 h and then treated with H_2_O_2_. Coverslips were stained with 10 μM Hoechst, placed in an incubation chamber with a controlled temperature and mounted on an Axiovert 200 M microscope equipped with a motorized stage. Nuclei were acquired with a 10x Fluar objective (Zeiss) and a CoolSnap HQ CCD camera. Twenty random fields were acquired with the random stage scan tools in MetaMorph and analyzed with the nuclei count application.

### Autophagy induction and inhibition

Twenty four hours after seeding, cells were extensively washed with PBS to remove any traces of the previous medium and then exposed to EBSS (Sigma-Aldrich) or to NH_4_Cl 2 mM for 3 h at 37°C and with controlled humidity and CO_2_.

### Autophagosomes count

HeLa cells were seeded as previously stated then transfected with a mix of LC3-EGFP cDNA in pcDNA3 and C19orf12-mKate2 or pmKate2 using the transfection procedure previously described. Thirty six hours after transfection cells were stained with Hoechst 1 μM then imaged with an Axiovert 200 M microscope equipped with a motorized stage and a CoolSnap HQ CCD camera. Ten random fields were acquired using a Zeiss 40X water immersion lens (N.A. 1.2). Images were then processed and autophagosomes counted using a custom made pipeline for the open source software Cell Profiler (Carpenter et al., 2006).

### Aequorin measurements

Cells grown on 13 mm round glass coverslips at 50% confluence were transfected with the mitochondria targeted aequorin. All aequorin measurements were carried out in 1 mM Ca^2+^ KRB buffer (NaCl 135 mM, KCl 5 mM, MgSO4 1 mM, K_2_HPO_4_0.4 mM, Glucose 5.5 mM, HEPES 20mM). Agonists and other drugs were added to the same medium, as specified in the figure legends. Experiments were stopped by lysing the cells with 100 μM digitonin in hypotonic Ca^2+^-rich solution (10 mM CaCl2 in H2O), thus discharging the remaining aequorin pool. The light signal was collected and calibrated into [Ca^2+^] values, as previously described (Bonora et al., 2013).

### Molecular modeling

The prediction of the transmembrane region has been carried out by MEMSAT3 (Jones, 2007) and its secondary structure propensity by McGuffin et al. (2000). The sequence of the predicted soluble regions of *C19orf12* (*C19orf1_1−40_*and *C19orf12_80−151_*) was used as a target sequence for homology modeling. The model was obtained by Modeller version 9.11 (Eswar et al., 2006) using the structure of its closer homolog, i.e., the Mg^2+^ transporter belonging to the MgtE class isolated from *Thermus thermophilus* (PDB entry 2yvy, chain A, residues 31–134, resolution 2.30 Å, (Hattori et al., 2007) as a template. C19orf12 shares 26% of sequence identity and 56% of sequence similarity with the template. The guide alignment for the prediction has been derived by HHPred (Söding et al., 2005) and then manually corrected to improve the match between the secondary structural elements of the template and the predicted secondary structural elements of the target, as well as to improve local sequence identity (Supplementary Figure 3). Model quality has been evaluated by AIDE program (Mereghetti et al., 2008).

## Results

### Wild-type and mutants C19Orf12 sub-cellular localization in native conditions

Prediction based on the amino acid sequence of human C19orf12 and fractionation experiments indicated that it was a mitochondrial membrane-bound 17-kDa protein (Hartig et al., 2012).

**Figure 1:**
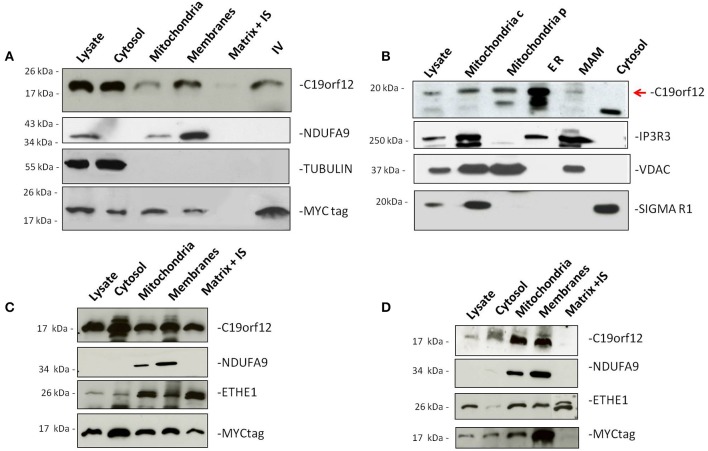
**Subcellular localization of wild-type and mutant C19orf12**. HeLa cells transfected with wild-type C19orf12^MYC^ construct **(A)** and mutant versions G58S^−^C19orf12^MYC^ construct **(C)**, and Q96P^−^C19orf12^MYC^ construct **(D)**, were harvested to obtain mitochondria and other fractions. Equal amount of proteins (30 μg) from each fraction were resolved by SDS-PAGE and immunostained with antibodies against C19orf12 and MYC to specifically detect protein sub-localization. Anti-NDUFA9, TUBULIN, and ETHE1 were used as control of mitochondrial membranes, cytosol fractions and mitochondrial matrix respectively. **(B)** Detection of naïve C19orf12 (red arrow) by immunoblotting in HEK 293 cells fractionation. The lower band is probably an unspecific signal. Mitochondria c, crude mitochondria; Mitochondria p, pure mitochondria; ER, endoplasmic reticulum; MAM, mitochondria-associated membrane. IS, Intermembrane space. IV, *In vitro* translation product. Anti-IP3R, VDAC, and Sigma-1R were used as ER, mitochondria and MAM markers respectively.

To demonstrate sub-cellular localization we performed Western-blot analysis on HeLa cells transfected with MYC-tagged *C19orf12* cDNA. Western-blot analysis showed that wild-type *C19orf12* gene product was present into the mitochondrial membranes but also in the lysate and cytosol (Figure 1A).

Because of its putative function in lipid metabolism (Hartig et al., 2012) we reasoned that the protein could have additional sub-cellular localizations. To demonstrate this we isolated different HEK293 fractions containing: crude mitochondria, pure mitochondria, membrane associated mitochondria (MAM), and ER.

Western-blot analysis of the different sub-cellular fractions using a specific C19orf12 antibody, indicated that the wild-type protein was present in both mitochondria and ER (Figure 1B) under naïve condition. Moreover, a small fraction of the protein was also detected in the MAM, which represent physical association between mitochondria and endoplasmic reticulum important for the transport of phospholipids (Patergnani et al., 2011; Marchi et al., 2014).

Antibodies specific to proteins known to be located into different sub-cellular compartments were used as controls. In particular, Inositol 3 Phosphate receptor 3 (IP3R3) was used as marker of ER, tubulin as marker of cytoplasm and the VDAC as marker for mitochondria.

To understand the localization of the mutant C19orf12 proteins, we performed Western-blot analysis on HeLa cells transfected with *C19orf12* versions, carrying the point mutations G58S and Q96P. The level of overexpression of Myc-tagged versions in relation to endogenous C19orf12 was evaluated by Real-time PCR and an histogram is reported in Supplementary Figure 1.

In the presence of G58S mutation, located in the predicted transmembrane domain, the mutant protein is also found in the mitochondrial matrix (Figure 1C). On the contrary the Q96P mutation has no effect on the localization of the protein, which is mainly present in the mitochondrial membranes (Figure 1D) as observed for the wild-type protein.

### Wild-type and mutants C19Orf12 live imaging analysis

To further corroborate the data obtained by Western-blot we performed experiments of live imaging in cells transfected with mkate2-tagged wild-type C19orf12, G58S, and Q96P mutant versions.

**Figure 2:**
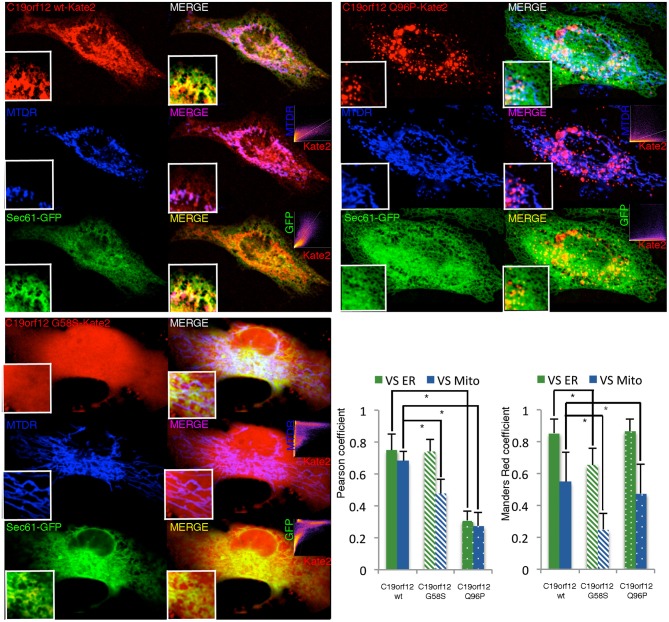
**Intracellular localization of wild-type and mutant C19orf12-mKate2 fusion protein**. Representative HeLa cells overexpressing wild-type C19orf12-mKate2 (red signal) or mutant variants G58S and Q96P. C19orf12-mKate2 colocalization with mitochondria (blue signal) or ER (green signal) is represented by two colors image merging of kate2 vs. mitochondrial (magenta signal) or vs. ER (yellow signal). For each merging the relative colocalization scatterplot is inserted as inset on bottom right corner. Analysis of colocalization is represented by Pearson's coefficient (indicating the correlation between mKate2 and mitochondria or ER signals) and by the Mander's Red coefficient (representing the proportion of mKate2 signal overlapping with mitochondria or ER). Bars: S.E.M., ^*^p < 0.05.

Cells transfected with the wild-type displayed a network-like intracellular staining with small tubular structures resembling the ER tubules and thicker structures similar to mitochondria (Figure 2). This localization was confirmed by confocal colocalization microscopy. mKate2 signal in fact display significant colocalization with the ER marker GFP–Sec61-β and also with the mitochondrial marker mitotracker Deep Red (as indicated by the high values of Pearson's and Mander's coefficients representing respectively the correlation between the two signals and the proportion of mKate2 signal overlapping with mitochondria or ER).

The G58S presented with a predominant cytosolic distribution that generates asymmetric behaviors in the colocalization indexes (Figure 2). Differently from what observed with the wild-type chimera, the Q96P displayed a vesicles pattern with a partial co-localization vs. the mitochondrial and ER compartments (as displayed by a reduction in the Pearson's coefficients) (Figure 2). Overall, the colocalization experiment confirms the data obtained by western-blot analysis (Figure 1) on different sub-cellular fractions.

### Response to oxidative stress

**Figure 3:**
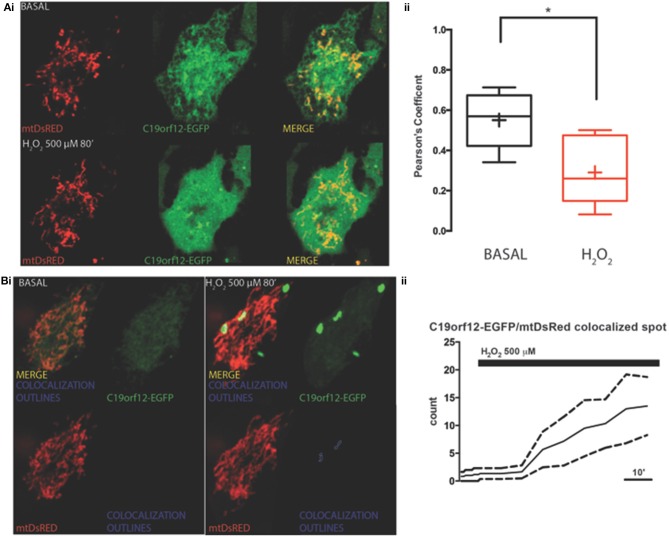
**Redistribution of C19orf12 during oxidative stress**. **(Ai)** Representative images of HeLa cells overexpressing the C19orf12-EGFP fusion protein and the mitochondrial marker mtDsRED before (upper panel), and after (lower panel) exposure to H_2_O_2_ 500 μM. **(ii)** Quantitative analysis of EGFP and DsRED signal before and after oxidative stress (cross, average; line, median; box, 25 and 75 percentile; bars, max and min value, *n* = 8, ^*^p > 0.05). **(Bi)** Representative distribution of C19orf12-EGFP fusion protein in HeLa cells displayed with low contrast and the mitochondrial marker mtDsRED before (left panel) and after (right panel) exposure to H_2_O_2_ 500 μM. **(ii)** Quantitative analysis of C19orf12-EGFP aggregates colocalizing with the mtDsRED signal during challenging with H_2_O_2_ 500 μM (continuous line, mean; dashed lines, S.E.M., *n* = 8).

To test response to oxidative stress we treated cells transfected with wild-type and mutant C19orf12 GFP-tagged versions, with 500 μM H_2_O_2_ for 80 min and we followed the cellular localization of the protein by live imaging during time. After 30 min from H_2_O_2_ addition, we observed that the wild-type changed its localization pattern from reticular to cytosolic and generated bright aggregates in proximity to the mitochondrial network (Figure 3). In addition, after persistent exposure to oxidative stress, it generates bright aggregates that partially colocalize with mitochondrial network (Figure 3B).

**Figure 4:**
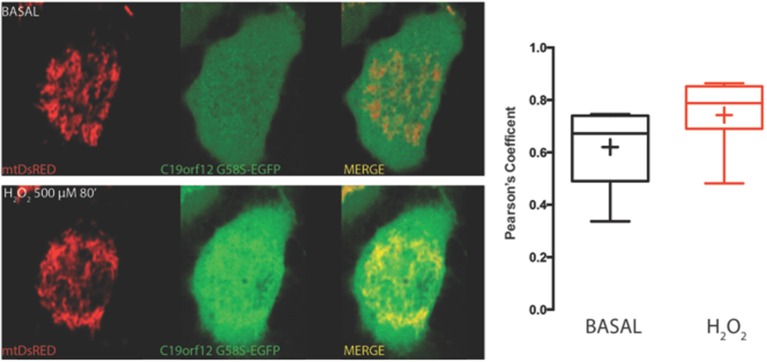
**Redistribution of C19orf12 G58S mutant during oxidative stress**. Representative HeLa cells overexpressing the C19orf12 G58S-EGFP fusion protein and the mitochondrial marker mtDsRED before (upper panel) and after (lower panel) exposure to H_2_O_2_ 500 μM. Quantitative analysis of EGFP and DsRED signal before and after oxidative stress (cross, average; line, median; box, 25 and 75 percentile; bars, max and min value, *n* = 8) is shown on the right.

**Figure 5:**
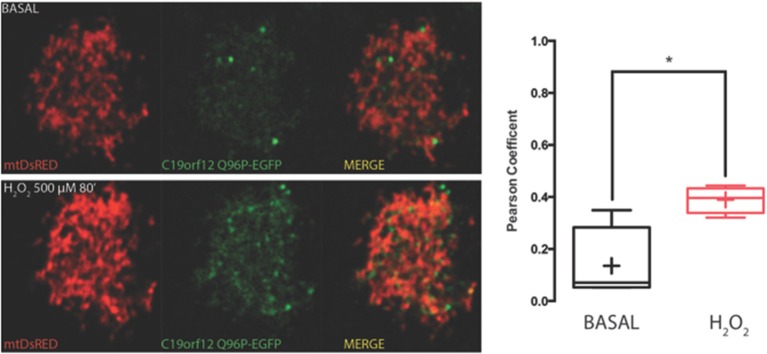
**Redistribution of C19orf12 Q96P mutant during oxidative stress**. Representative HeLa cells overexpressing the C19orf12 Q96P-EGFP fusion protein and the mitochondrial marker mtDsRED before (upper panel) and after (lower panel) exposure to H_2_O_2_ 500 μM. Quantitative analysis of EGFP and DsRed signal before and after oxidative stress (cross, average; line, median; box, 25 and 75 percentile; bars, max and min value, *n* = 8, ^*^, *p* > 0.05) is shown on the right.

On the contrary, both mutant G58S (Figure 4) and Q96P (Figure 5) versions display minor redistribution as indicated by the variation in the Pearson's coefficient. Only the mutant Q96P displayed a significant increase in Pearson's coefficient that remains in any case lower then 0.5, usually considered as threshold for a relevant correlation (Bolte and Cordelières, 2006) suggesting that this mutant increases its cytosolic distribution without affecting dramatically its mitochondrial localization.

**Figure 6:**
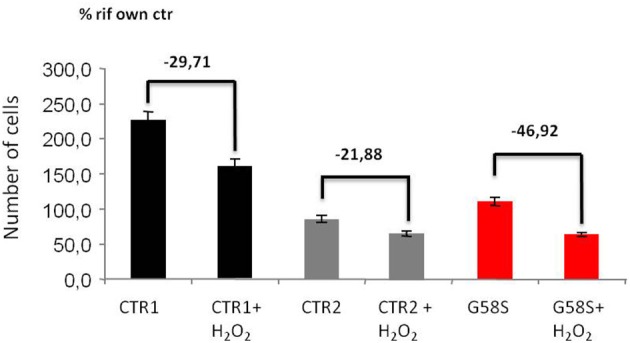
**Fibroblasts with C19orf12 mutation are more sensitive to cell death**. Human fibroblasts were treated with Hydrogen Peroxide (2 mM H_2_O_2_ for 5 h). Apoptosis was evaluated using an automated nuclei count analysis. Numbers above bars indicate the percentage of cell death in the presence of H_2_O_2_ as compared to the corresponding untreated sample.

We also tested apoptotic cell death after H_2_O_2_ treatment and we observed that fibroblasts derived from the patient carrying the G58S change were more sensitive to treatment and showed a high percentage of cells death as compared to two control fibroblasts (Figure 6). We could not test the Q96P mutation since patient' fibroblast were not available.

### Analysis of mitochondrial Ca^2+^ homeostasis

**Figure 7:**
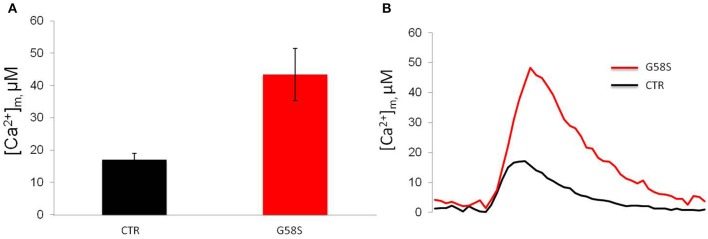
**Fibroblasts with C19orf12 mutation displayed increased Ca^2+^ mobilization**. **(A)** Mitochondrial Ca^2+^ responses to agonist stimulation (100 μM ATP) measured in human fibroblasts. Graphs show quantification of mitochondrial Ca^2+^ from three independent experiments. **(B)** Representative traces of Ca^2+^ responses. CTR [Ca^2+^]_m_ peak 17. 0 ± 1.96 μM; G58S [Ca^2+^]_m_ peak 43. 4 ± 8.09 μM.

In order to measure mitochondrial Ca^2+^ handling (Marchi et al., 2014) in controls and patient-derived fibroblasts we carried out mitochondrial [Ca^2+^] ([Ca^2+^]_m_) measurements using the mitochondrial-targeted aequorin probe (Bonora et al., 2013). To this end we stimulated the cells with an agonist, ATP, acting on receptors coupled, through Gq proteins, to the production of inositol 1,4,5 trisphosphate (IP3) and in turn to the opening of the IP3 receptor. Both in control and cells harboring the G58S mutation, ATP stimulation caused a rapid rise in [Ca^2+^]_m_ followed by a gradually declining sustained plateau. In patient-derived fibroblasts, the [Ca^2+^]_m_ increases evoked by stimulation with ATP were significantly greater than in controls (Figure 7).

### Evaluation of autophagy

**Figure 8:**
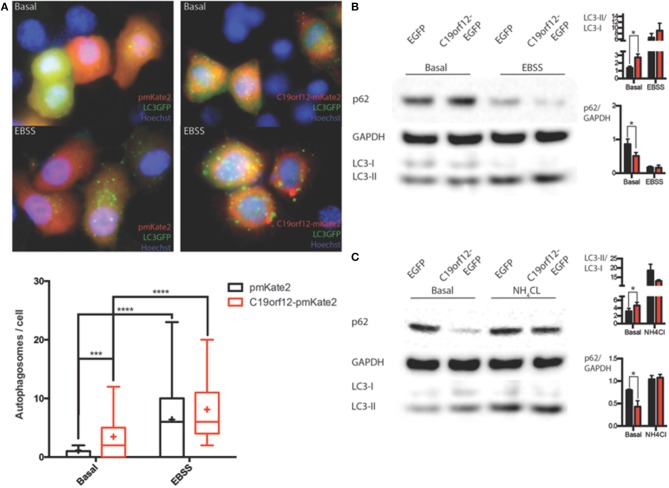
**Analysis of autophagy during C19orf12 wild-type overexpression**. Representative images of HeLa cells overexpressing C19orf12-mKate2 or empty pmKate2 simultaneously with the autophagic marker LC3-EGFP **(A)** in basal condition or after exposure to EBSS. In the lower panel quantification of autophagosome counts is displayed (cross, average; line, median; box, 25 and 75 percentile; bars, max and min value, *n* = 8, ^***^p > 0.005, ^****^p > 0.001). **(B)** Representative western blot analysis of autophagic markers LC3 and p62 in HeLa cells overexpressing C19orf12-EGFP or EGFP empty vector as control in basal condition or after exposure to EBSS (bars, S.E.M.; *n* = 4; ^*^p > 0.05). **(C)** Representative western blot analysis of autophagic marker LC3 and p62 in HeLa cells overexpressing C19orf12-EGFP or EGFP empty vector as control in basal condition or after exposure to NH_4_Cl 2mM (bars, S.E.M.; *n* = 4; ^*^p > 0.05).

In order to understand the nature of the aggregates formed by the wild-type protein, surrounding mitochondria, we performed colocalization study using the specific autophagy marker LC3. Confocal live imaging of LC3 vesicles and C19orf12 displayed that the C19orf12 redistribution induced by oxidative stress inversely correlated. Indeed while H_2_O_2_ induced aggregates formation it also reduces the amount of LC3 vescicles (Supplementary Figure 2A). The amount of colocalized dots increased about 50% in response to H_2_O_2_ exposure (Supplementary Figure 2Aiv). Overall 3D confocal microscopy display that only a minor proportion of LC3-EGFP puncta co-localize with C19orf12-mKate2 aggregates after H_2_O_2_ exposure (Supplementary Figures 2B). Nonetheless, the effect of C19orf12 on autophagy was evaluated. Coexpression of the autophagic reporter LC3-EGFP and of C19orf12-mKate2 displays a higher amount of EGFP punctae (autophagosomes) compared to cells expressing the autophagic marker with the pmKate2 empty vector (Figure 8A). This data was corroborated by analysis of endogenous LC3 marker. Overexpression of the EGFP tagged wild type C19orf12 induce the conversion of the autophagic marker LC3 heavy form (LC3I) to the light form (LC3II) compared to cells transfected with the empty EGFP vector, indicating the elevation of basal autophagic levels (Figure 8B). In both assays the LC3 conversion was further stimulated when inducing autophagy by exposing cells to EBSS medium. The promoted conversion of LC3 induced by C19orf12-EGFP overexpression did not appear as a blocked autophagic flux. In fact, overexpression of this plasmid was sufficient to induce a reduction of the autophagic marker p62. This protein is usually required for autophagosome formation and its levels are expected to decrease during autophagy due to degradation of autophagosome content (Klionsky et al., 2012). Indeed treatment with NH_4_Cl lead to impaired acidification of autophagosomal content and inhibition of autophagosome degradation, with concomitant LC3 conversion and p62 accumulation (Figure 8C).

**Figure 9:**
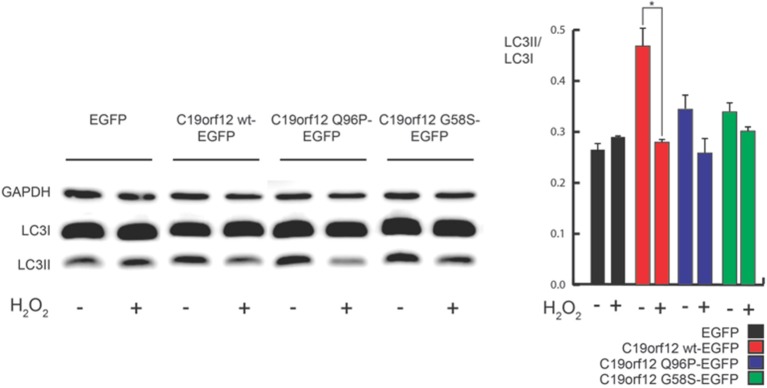
**Analysis of autophagy during C19orf12-EGFP wild-type and mutants overexpression**. Representative western blot analysis of autophagic marker LC3 in HeLa cells overexpressing C19orf12-EGFP, C19orf12 G58S-EGFP, C19orf12 Q96P-EGFP, or EGFP empty vector as control (left panel). Densitometry of light LC3 chain (LC3II) bands normalized on heavy LC3 chain (LC3I) bands is shown (right panel). Analysis was performed in basal condition or after stimulation with 500 μM H_2_O_2_ for 80 min (bars, S.E.M.; *n* = 4, ^*^p > 0.05).

Interestingly, H_2_O_2_ treatment inhibits the observed effect on autophagic levels (Figure 9) but caused the relocalization of C19orf12 (Figure 3). In support of this observation, the overexpression of the vectors carrying the mutant forms G58S and Q96P was unable to induce LC3 conversion suggesting a localization dependent role for C19orf12 in regulation of autophagy (Figure 9).

### In silico analyses

We carried out both secondary structure prediction of the full *C19orf12* sequence and modeling of the predicted soluble region (*C19orf12_1−40/81−151_*) to both understand functional and structural properties of the wild-type protein and the effects of the mutations. C19orf12 was predicted to contains two α-helices located in the trans-membrane (TM) region (Supplementary Figure 3) rich in glycine residues, of which several have been found mutated in MPAN patients: G58S (Panteghini et al., 2012), G53R, G65E, G69R, (Landouré et al., 2013) *C19orf12* contains in the transmembrane helix glycine zipper motifs, (GxxxGxxxG) (Kruer et al., 2014). The most significant glycine zipper patterns in proteins that have been reported so far are (G,A,S)XXXGXXXG and GXXXGXXX(G,S,T) (Kim et al., 2005). The first motif (AXXXGXXXG) corresponding to the sequence _50_AFVG**G**LVG**G**_58_ where both G53R and G58S mutations occur is located in close proximity to the first TM α-helix. The second motif _61_GLAV**G**GAV**G**GLL**G_73_** is longer and contains two of those repeats, with the mutations G65E and G69R. The N- and C-terminal residues (C19orf12_1−41/77−151_) are predicted to rearrange in a soluble three-dimensional (3D) domain homologous to the N-regulatory domain of the bacterial Mg^2+^ transporters of the MgtE (Maguire, 2006; Payandeh et al., 2013). In the MgtE transporters, this domain forms a right-handed superhelical structure that includes 10 helices per two turns.

**Figure 10:**
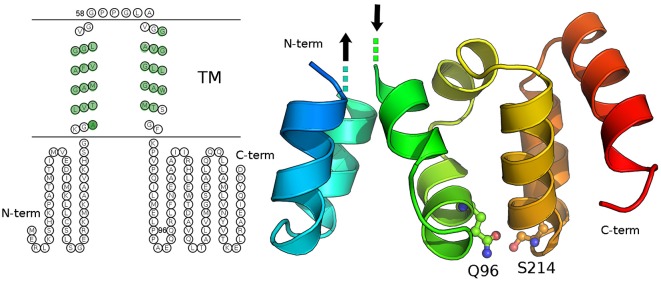
**Secondary and tertiary structure of C19orf12. Left panel:** The prediction of transmembrane regions carried out with MEMSAT is illustrated and the residues of the transmembrane region, which are predicted in helical structures by PSI-Pred are highlighted in green. **Right panel**: The three-dimensional (3D) model of the C19orf12 domain homologous to the MgtE N-domain is shown in cartoon. The mutation site Gln96 and the residues Ser214 are shown as sticks and spheres. The protein is shown with shade of colors from blue to red, from the N- to the C-terminal extremity, respectively. The dots and the arrows illustrate the regions that are expected to connect the domain to the membrane. Gly58 is not reported since a reliable 3D model is not available for the transmembrane domain.

Our model does not provide a reliable prediction for the first 14 amino acids of *C19orf12*, which would correspond to the first two helices of the right-handed superhelical motif due to poor sequence similarity with known structures of MgtE-like transporter. The rest of the domain is well conserved with respect to the bacterial homologs and in this region the Q96P is located in the middle of one of the α helices (corresponding to the α6 of the bacterial N-domain) and well packed within the domain (Figure 10). It is a mutation from a polar residue to a proline, which is a well-known helix-breaker. *FoldX* (Schymkowitz et al., 2005) energy was used to estimate the free-energy changes upon Q96P mutation. In particular, the changes in protein stability upon the mutation were estimated as the difference (ΔΔG) between the free energies of unfolding (ΔG) of the mutant and the wild-type variant. ΔΔG values above 1.6 kcal/mol are expected to significantly affect stability because they correspond to twice the standard deviation of *FoldX* (Schymkowitz et al., 2005) Q96P mutation is predicted to impair protein stability of 5.4 ± 0.3 kcal/mol, (Guerois et al., 2002) suggesting a loss of protein stability upon this mutation In the model structure, Gln96 is predicted to be involved in side-chain hydrogen bonds, as the one with Ser124, located in the loop than connect helices α8 and α9 (Figure 10). The structural rearrangement caused by Q96P mutation might influence the network of polar interactions mediated by Gln95.

## Discussion

*C19orf12* was reported to code for a mitochondrial membrane protein probably involved in lipid metabolism (Hartig et al., 2012). We here demonstrated that C19orf12 protein is not only present in mitochondria but also in ER and MAM. These are zones of close contact between ER and mitochondria, which support communication between the two organelles as concerning lipid transfer and Ca^2+^ ions exchange. This activity regulates several processes including: ER chaperone-assisted folding of newly synthesized proteins, modulation of mitochondria-localized dehydrogenases involved in ATP-producing Krebs cycle reactions, activation of Calcium-dependent enzymes that execute cell death programs (Berridge, 2002). We observed that the G58S mutant protein was also present into the mitochondrial matrix and we reasoned whether this different sub-cellular localization could also affect its functionality. The C19orf12 protein belongs to the clan of glycine zipper containing membrane domains (Kim et al., 2005). The majority of C19orf12 mutations are clustered in a functional region, which is crucial for this superfamily of proteins and is characterized, in the TM regions, by long and repeated glycine-zipper motifs, generally GxxxGxxxG. This is a common motif in several multimeric known membrane channel structures, where the glycine faces are in direct contacts (Kim et al., 2005). Notably, this pattern is statistically over-represented in membrane proteins in general (Kim et al., 2005). It has been indeed proposed to be the driving force for right-handed packing against a neighboring helix. It has also been suggested to play a crucial role in gating mechanisms (Kim et al., 2005). The glycine zipper motifs of C19orf12 suggest that they are involved in the interaction between the two TM helices of this protein, even if we cannot rule out also a putative involvement in homo-dimerization. Mutations of the glycines of the glycine-zipper motif to charged or polar residues, as observed in the mutant C19orf12 patients, were likely to impair the correct localization of the protein in the membrane. These bioinformatics predictions fully agreed with the experimental data obtained by western-blot investigation. Indeed, we observed a prevalent cytosolic localization of the mutant G58S protein, while the fraction present in mitochondria was also found in the matrix, indicating that the protein was not tightly bound to the membrane.

Interestingly, glycine zipper motifs have been found in Ap and PrP, which are associated with Alzheimer's and prion diseases. A neuropathological hallmark of both Alzheimer's disease and spongiform encephalopathies includes the formation of deposits in the brain such as amyloid plaques, glial responses and neurofibrillary tangle (Jeffrey, 2013). It is important to notice that histopathological examination of the brain from a single MPAN patient also revealed the presence of Lewy bodies, tangles, spheroids, and tau pathology (Hartig et al., 2012), suggesting a possible common pathological role for the motif in these neurodegenerative disorders.

We predicted that C19orf12 soluble domain is homologous to the N-terminal regulatory domain of bacterial MgtE transporters. The comparison of the Mg^2+^-free and –bound structures of a MgtE transporter (Hattori et al., 2007) and NMR experiments (Imai et al., 2012) showed a rearrangement of the N-domain upon Mg^2+^-interaction. Moreover, MgtE variants lacking the N-terminal subdomains showed a reduced Mg^2+^-dependent inhibition and an increased open probability, implicating this subdomain in MgtE function and regulation (Hattori et al., 2009), acting as a sensor of Mg^2+^ concentration. In eukaryotic organisms, MgtE-like genes belong to the SCL41 family and their precise role is unknown (Fleig et al., 2013; Schweigel-Röntgen and Kolisek, 2014). Interestingly, the N-terminal regulatory domain of bacterial MgtE is missing in SLC41-A1, thus implying that the eukaryotic transporters evolved different mechanisms of regulation (Schweigel-Röntgen and Kolisek, 2014). The homology of the soluble portion of C19orf12 with this bacterial subdomain, and its localization in membrane, would support a function for C19orf12 as a regulatory domain of eukaryotic MgtE-like proteins, different from SLC41-A1.

*In silico* investigation of the Q96P predicted for this mutation to cause loss of side-chain mediated hydrogen bonds and to affect the correct architecture of a central α-helix in the 3D structure of the C19orf12 soluble domain homologous to the N-terminal regulatory domain of bacterial MgtE transporters. This suggests a possible role of the α domain in the interaction and regulation of C19orf12 protein with human MgtE-like transporters, acting as a regulatory protein.

Interestingly, deficiency of systemic and intracellular magnesium (Mg) has long been suspected to contribute to the development and progression of Parkinson's and other neurodegenerative diseases, although the molecular mechanism is still unknown (Kolisek et al., 2013).

To gain insight into the pathogenic role of C19orf12 in MPAN we performed *in vitro* investigations by challenging the cells with stressful conditions and by evaluating the response of the wild-type and mutant C19orf12 proteins.

We proved that the wild-type C19orf12 protein was able to respond to oxidative stress by enriching its cytoplasmic localization and forming aggregates, which partially co-localized with mitochondria. On the contrary, both C19orf12 mutant proteins were insensitive to oxidative stress and did not form aggregates. In light of the recent observation, that the ER–mitochondria contact sites are important in autophagosome formation (Hamasaki et al., 2013) we proposed a putative role for C19orf12, in control of autophagy. In support of this hypothesis we observed that overexpression of wild-type C19orf12 resulted in conversion of autophagic marker LC3 and reduction of levels of p62. On the contrary, induction of delocalization by oxidative stress results in reduction of autophagy LC3 conversion. Interestingly, the overexpression of mutants, unable to properly gain its intracellular localization, fails to promote autophagy induction and levels of basal autophagy remain unchanged during exposure to oxidative stress.

Live imaging suggested that delocalization of C19orf12 appears related to existence of LC3-vescicles. Indeed the progressive accumulation of C19orf12 in cytoplasm and its accumulations in aggregates were concomitant with the reduction in number of LC3-EGFP vesicles. Furthermore, the amount LC3-EGFP vescicles co-localizing with C19orf12 was extremely low. Since it was reported that the marker LC3-EGFP could produce non-autophagosome related aggregates (Kuma et al., 2007), also C19orf12 aggregates co-localizing with LC3 puncta have dimension larger then 1.5 μm (average feret 1.98 μm, SEM 0.17, *n* = 9) suggesting that these were not autophagosomes. These results would therefore suggest that the C19orf12 is contemporary able to exert an inhibitory effect on apoptosis induction and a stimulatory effect on autophagy. The loss of autophagy induction observed after mutants overexpression and the increased sensitivity to apoptosis in patients-derived fibroblasts carrying mis-localized mutants, suggests that C19orf12 can induce protective autophagy at the expense of apoptosis and that this effect could be dependent on its intracellular localization.

These results suggest that C19orf12 could be involved in removal of dysfunctional mitochondria by selective autophagy (in a fashion independent on aggregates formation). Considering that MPAN disease mainly affects the brain, it is well possible that neurons carrying C19orf12 mutations, could accumulate altered mitochondria which can't be removed because of the presence of C19orf12 mutations, and could degenerate and/or eventually die. Nonetheless the present results about the role of C19orf12 in regulation of autophagy will require more detailed studies in future.

Finally, we also observed high levels of mitochondrial Ca^2+^ in fibroblasts derived from patients as compared to control, suggesting that the mutations altering the intracellular distribution of C19orf12 is detrimental for proper mitochondrial function and Ca^2+^ homeostasis. As a consequence, patient-derived fibroblasts were more sensitive to Ca^2+^ dependent apoptotic stimuli like H_2_O_2_ induced death as compared to control fibroblasts.

We here demonstrated that C19orf12 protein involved in NBIA is located in mitochondria and also present in the ER as previously reported (Landouré et al., 2013), and MAM. Moreover, we proposed a role for this protein as a sensor of mitochondrial damage. We also demonstrated that patients-derived fibroblasts accumulated high levels of mitochondrial Ca^2+^ and were more prone to oxidative stress induced apoptosis. Altogether these data shed new light in the field of NBIA focusing the attention on the role of mitochondria-ER connection in the transfer of essential lipids, in calcium metabolism and in autophagosome formation (Hamasaki et al., 2013), which are fundamental for the maintenance of cellular homeostasis and for determination of cell fate under pathological condition. A role of MAM has been recently proposed in another neurodegenerative disorder that is Alzheimer's disease (Schon and Area-Gomez, 2010) with the demonstration that presenilin 1 and 2 are predominantly located into these specialized structures. It is well possible that proteins such as presenilin 1 and 2, and C19orf12, can shuttle between different sub-cellular compartments depending on the cells status. Moreover, molecular homology modeling suggested a putative role for C19orf12 in regulation of magnesium transport. Magnesium homeostasis is crucial for learning and memory and has a positive effect on synaptic plasticity and density (Barbagallo et al., 2009; Slutsky et al., 2010). Moreover, magnesium and calcium work together to modulate ion channels, which open in response to nerve impulses triggering neurotransmitter release (Slutsky et al., 2004; Bardgett et al., 2005). These observations are particularly relevant in the context of a neurodegenerative disease such as NBIA, but dedicated experiments are required to further demonstrate this hypothesis.

### Conflict of interest statement

The authors declare that the research was conducted in the absence of any commercial or financial relationships that could be construed as a potential conflict of interest.

## Supplementary material

Supplementary Figure 1 **Real-time PCR to evaluate expression level of C19orf12-MYC**. Expression level of overexpressed C19orf12-MYC versions (wild-type, G58S, Q96P, respectively) evaluated as fold-change in comparison to endogenous C19orf12.

Supplementary Figure 2 **(Ai)** Representative behavior of C19orf12-mKate2 aggregates and LC3-EGFP before (left panel) and after (right panel) exposure to H_2_O_2_ 500 μM. Quantitative analysis of channel independent spot counts: LC3-EGFP vescicles **(ii)**, mkate2 aggregates **(iii)**, and colocalized spot **(iv)** during challenging with H_2_O_2_ 500 μM (continuous line: mean, dashed lines: S.E.M., *n* = 8). ** (B)** Representative 3D images of autophagic vesicles in presence of H_2_O_2_ 500 μM. **(i)** LC3-EGFP (green), **(ii)** C19orf12-mKate aggregates (red), and **(iii)** colocalization signal (yellow). Multiple orthogonal view of the merged signal are displayed and marked by sequential numbering. **(iv)** Percentage of C19orf12-mKate2 aggregates colocalizing with LC3 vescicles **(v)** LC3 puncta co-localizing with mKate2 aggregates (cross, average; line, median; box, 25 and 75 percentile; bars, max and min value, *n* = 12).

Supplementary Figure 3 **Guide alignment for homology modeling**. The sequence alignment between the target (C19orf12) and the template (PDB entry 2yvy, chain A) is shown as derived by the HHPred multiple sequence alignment upon manual correction (see Materials and Methods). “^*^” and “.” indicates identical and similar residues, respectively.
