# Supplementary figures and images for: Mutations of C19orf12, coding for a transmembrane glycine zipper containing mitochondrial protein, cause mis-localization of the protein, inability to respond to oxidative stress and increased mitochondrial Ca2+
Source: Front Genet. 2015 May 19;6:185. doi: 10.3389/fgene.2015.00185 (PMC4470416; doi:10.3389/fgene.2015.00185)

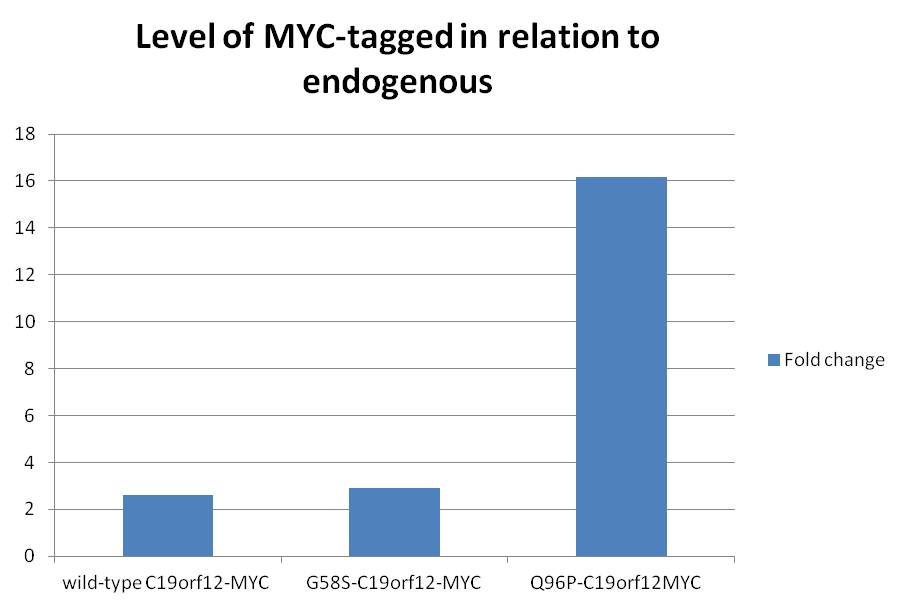

Supplement: Supplementary Figure 1 — Real-time PCR to evaluate expression level of C19orf12-MYC. Expression level of overexpressed C19orf12-MYC versions (wild-type, G58S, Q96P, respectively) evaluated as fold-change in comparison to endogenous C19orf12. [file Image1.JPEG]

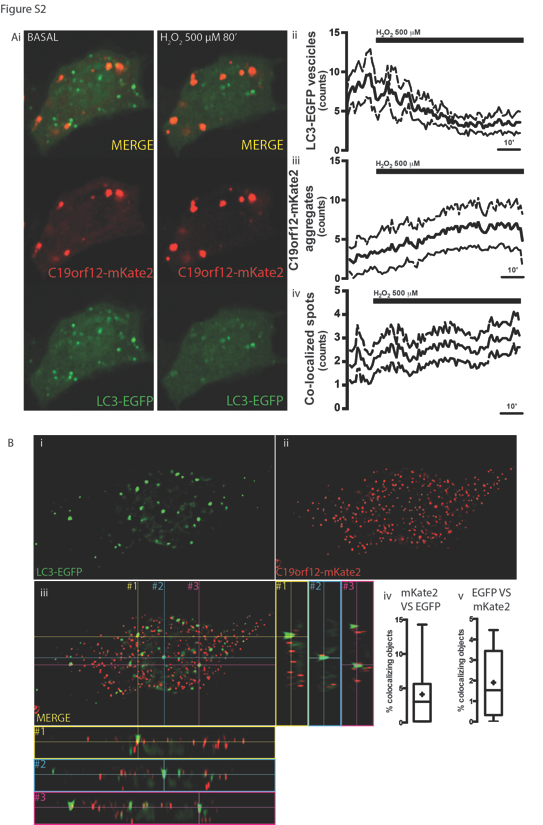

Supplement: Supplementary Figure 2 — (Ai) Representative behavior of C19orf12-mKate2 aggregates and LC3-EGFP before (left panel) and after (right panel) exposure to H2O2 500 μM. Quantitative analysis of channel independent spot counts: LC3-EGFP vescicles (ii), mkate2 aggregates (iii), and colocalized spot (iv) during challenging with H2O2 500 μM (continuous line: mean, dashed lines: S.E.M., n = 8). (B) Representative 3D images of autophagic vesicles in presence of H2O2 500 μM. (i) LC3-EGFP (green), (ii) C19orf12-mKate aggregates (red), and (iii) colocalization signal (yellow). Multiple orthogonal view of the merged signal are displayed and marked by sequential numbering. (iv) Percentage of C19orf12-mKate2 aggregates colocalizing with LC3 vescicles (v) LC3 puncta co-localizing with mKate2 aggregates (cross, average; line, median; box, 25 and 75 percentile; bars, max and min value, n = 12). [file Image2.TIFF]
