# Supplementary material for: Mutations of C19orf12, coding for a transmembrane glycine zipper containing mitochondrial protein, cause mis-localization of the protein, inability to respond to oxidative stress and increased mitochondrial Ca2+
Source: Front Genet. 2015 May 19;6:185. doi: 10.3389/fgene.2015.00185 (PMC4470416; doi:10.3389/fgene.2015.00185)
Supplement: Supplementary Figure 3 — Guide alignment for homology modeling. The sequence alignment between the target (C19orf12) and the template (PDB entry 2yvy, chain A) is shown as derived by the HHPred multiple sequence alignment upon manual correction (see Materials and Methods). “*” and “.” indicates identical and similar residues, respectively. [file Image3.PDF]

```
ss_pred       CCHHHHHHHHHHHHHhhccchhhhhhCC-CcHHHHHHhCCHHHHH-HHHH----HHHHHHhcCCcccHHH-HHHHHhcCHHH
C19orf12 14   IMVEDIMKLLCSLSGERKMKAAVKHSG-KPVPQILMELPPAEQQ-RLFN----EAAAIIRHLEWTDAVQ-LTALVMGSEAL 87
              *  .*.. *   * **..  ..      * . ..*  * * **   * .     .  *.  *   *... *.*.     *
2yvy_A    31  IHPQDLLALWDELKGEHRYVVLTLLPKAK-AAEVLSHLSPEEQAEYLKTLPPWRLREILEELSLDDLADALQAVRKEDPAY 110
ss_dssp       CCHHHHHHGGGGSCHHHHHHHHHHHSCHHH-HHHHHHTSCHHHHHHHHHHHSCHHHHHHHHHHHSCHHHHHHHHHHHHHHHCHHH

ss_pred       HHHHHHHHHHHHHHHHHhceeccCC
C19orf12  88  QQQLLAMLVNYVTKELRAEIQYDD 111
               *.*  .*    . *. * ..*..
2yvy_A   111  FQRLKDLLDPRTRAEVEALARYEE 134
ss_dssp       HHHHHHHSCHHHHHHHHHHHHHSCT
```
